# Mitapivat‐Associated Adverse Effects and Potential Mechanistic: Insights From Real‐World Data

**DOI:** 10.1111/jcmm.70893

**Published:** 2025-10-13

**Authors:** Guojun Liang, Qiong Liu

**Affiliations:** ^1^ Huadu District People's Hospital of Guangzhou Guangzhou Guangdong China

To the Editor,

Mitapivat, an oral activator of pyruvate kinase (PKR), was approved by the FDA in 2022 for adults with pyruvate kinase deficiency (PKD) [1]. By enhancing PKR activity, it increases ATP production, stabilises red cell metabolism and reduces hemolysis and transfusion needs. Phase 3 trials, including ACTIVATE and ACTIVATE‐T, confirmed efficacy and tolerability, with adverse events mainly consisting of laboratory abnormalities and nonspecific symptoms [2, 3, 4]. Yet, its safety in broader populations remains incompletely defined. To investigate post‐marketing signals, we conducted a disproportionality analysis using the FDA Adverse Event Reporting System (FAERS).

We retrieved FAERS reports from January 2022 to June 2025 in which Mitapivat was the primary suspect drug. After FDA‐recommended deduplication, events were mapped to MedDRA version 26.0 preferred terms (PT) and system organ classes (SOC) [5]. The reporting odds ratio (ROR) was used as the primary measure of disproportionality, and supportive frequentist and Bayesian approaches (PRR, IC, EBGM) were applied to confirm the consistency of findings [6, 7, 8, 9].

Among 560 reports, most were non‐serious (82.7%) and submitted by consumers (82.0%), whereas only 8.0% were filed by health professionals. Nearly all originated from the United States (Table 1). The median time to onset was 134 days, highlighting delayed toxicities that may require prolonged clinical monitoring (Figure S1).

**TABLE 1:** Clinical characteristics of reports with Mitapivat from the FAERS database (January 2022 to June 2025).

Characteristics	Mitapivat induced AE reports (*n* = 560)	Case proportion, %
Number of events	Case number, *n*	
**Gender, *n* (%)**		**100.00**
Female	300	53.60
Male	218	38.90
Missing	42	7.50
**Age (years), *n* (%)**		**100.00**
< 18	1	0.20
18 ≤ and < 65	84	15.00
≥ 65	12	2.10
Missing	463	82.70
**Reported countries, *n* (%)**		**100.00**
USA	558	99.80
Other	2	0.20
**Outcomes, *n* (%)**		**100.00**
Non‐serious	463	82.70
Serious	97	17.30
Death	1	0.20
Disability	1	0.20
Hospitalisation	52	9.30
Life‐threatening	1	0.20
Other	505	90.20
**Reporters, *n* (%)**		**100.00**
Consumer	459	82.00
Health Professional	45	8.00
Pharmacist	2	0.40
Physician	54	9.60
**Reporting year, *n* (%)**		**100.00**
2022	86	15.40
2023	183	32.70
2024	191	34.10
2025	100	17.90

Disproportionality analysis yielded 41 preferred terms with statistically significant signal strength across 14 system organ classes (Table 2). Some findings, such as arthralgia, back pain and increased urate, were aligned with the prescribing information. However, new signals were detected. Hematologic and metabolic terms including blood iron increased (ROR: 397.67), iron overload (ROR: 153.86) and haemoglobin abnormal (ROR: 83.14) suggest disturbances in iron handling and erythropoiesis not emphasised in clinical trials. Because iron overload is common in PKD patients due to transfusion history, these associations may reflect a complex interaction between underlying disease and drug effect rather than direct causation.

**TABLE 2:** Distribution of adverse event reports by System Organ Class (SOC) for Mitapivat in the FAERS database.

SOC	PT (preferred term)	*N*	ROR (95% Cl)	IC (IC025)	PRR (*χ* ^2^)	EBGM (EBGM05)
Blood and lymphatic system disorders	Haemolysis	6	36.12 (16.18–80.65)	5.16 (1.49)	36 (203.38)	35.86 (16.06)
Cardiac disorders	Atrial fibrillation	9	3.61 (1.87–6.95)	1.85 (0.6)	3.6 (16.89)	3.6 (1.87)
	Cardiac disorder	6	2.82 (1.26–6.28)	1.49 (0.07)	2.81 (7.01)	2.81 (1.26)
Gastrointestinal disorders	Dry mouth	7	3.4 (1.62–7.15)	1.76 (0.36)	3.39 (11.83)	3.39 (1.61)
General disorders and administration site conditions	Fatigue	76	3.4 (2.7–4.28)	1.72 (1.34)	3.3 (123.17)	3.3 (2.62)
	Malaise	32	3.15 (2.22–4.47)	1.64 (1.04)	3.11 (46.1)	3.11 (2.19)
	Asthenia	27	2.8 (1.91–4.09)	1.47 (0.83)	2.77 (30.69)	2.77 (1.89)
	Therapy Non‐Responder	13	6.88 (3.99–11.88)	2.77 (1.5)	6.84 (64.82)	6.83 (3.96)
	Thirst	6	11.05 (4.96–24.65)	3.46 (1.09)	11.02 (54.61)	11.01 (4.94)
Hepatobiliary disorders	Jaundice	13	29.89 (17.31–51.63)	4.89 (2.51)	29.68 (359.19)	29.59 (17.13)
	Ocular Icterus	6	76.9 (34.38–172.01)	6.25 (1.6)	76.65 (444.12)	75.99 (33.98)
Infections and infestations	Nasopharyngitis	18	2.97 (1.86–4.72)	1.56 (0.75)	2.95 (23.21)	2.95 (1.85)
	Influenza	11	2.94 (1.62–5.32)	1.55 (0.5)	2.93 (13.97)	2.93 (1.62)
	Infection susceptibility increased	6	81.13 (36.27–181.49)	6.32 (1.6)	80.86 (468.95)	80.13 (35.82)
Investigations	Haemoglobin decreased	138	60.08 (50.47–71.51)	5.78 (5.05)	55.47 (7345.68)	55.13 (46.31)
	Blood iron increased	17	397.67 (244.08–647.89)	8.56 (3.41)	393.86 (6377.22)	377.08 (231.44)
	Haemoglobin abnormal	16	83.14 (50.71–136.33)	6.35 (3.12)	82.4 (1274.82)	81.65 (49.79)
	Blood pressure increased	10	2.25 (1.21–4.2)	1.17 (0.14)	2.25 (6.94)	2.25 (1.21)
	Laboratory test abnormal	8	10.25 (5.12–20.54)	3.35 (1.37)	10.21 (66.41)	10.2 (5.09)
Metabolism and nutrition disorders	Gout	4	8.5 (3.18–22.68)	3.08 (0.47)	8.48 (26.38)	8.47 (3.18)
	Iron overload	4	153.86 (57.19–413.9)	7.24 (0.98)	153.51 (595.73)	150.91 (56.1)
Musculoskeletal and connective tissue disorders	Arthralgia	43	3.26 (2.41–4.41)	1.68 (1.17)	3.2 (65.57)	3.2 (2.36)
	Back pain	33	5.59 (3.96–7.89)	2.46 (1.78)	5.5 (121.97)	5.5 (3.9)
	Myalgia	13	3.27 (1.89–5.64)	1.7 (0.71)	3.25 (20.31)	3.25 (1.88)
	Bone pain	13	9.49 (5.5–16.37)	3.23 (1.78)	9.42 (97.86)	9.41 (5.45)
	Osteoporosis	5	3.85 (1.6–9.26)	1.94 (0.2)	3.84 (10.51)	3.84 (1.6)
Nervous system disorders	Headache	40	2.58 (1.89–3.54)	1.35 (0.84)	2.55 (37.98)	2.55 (1.86)
	Dizziness	21	1.73 (1.13–2.66)	0.79 (0.12)	1.72 (6.43)	1.72 (1.12)
	Memory impairment	12	3.27 (1.85–5.77)	1.7 (0.67)	3.25 (18.75)	3.25 (1.84)
	Brain fog	11	10.06 (5.56–18.2)	3.32 (1.68)	10 (89.05)	9.99 (5.52)
	Disturbance in attention	11	8.99 (4.97–16.28)	3.16 (1.59)	8.94 (77.59)	8.94 (4.94)
Psychiatric disorders	Insomnia	14	2.31 (1.37–3.91)	1.2 (0.33)	2.3 (10.34)	2.3 (1.36)
	Sleep disorder	14	6.2 (3.66–10.49)	2.62 (1.45)	6.16 (60.5)	6.15 (3.64)
	Poor quality sleep	4	6.19 (2.32–16.51)	2.63 (0.31)	6.17 (17.34)	6.17 (2.31)
	Libido decreased	3	12.24 (3.94–38.02)	3.61 (0.24)	12.22 (30.87)	12.2 (3.93)
Reproductive system and breast disorders	Menstrual disorder	3	16.86 (5.43–52.39)	4.07 (0.32)	16.83 (44.6)	16.8 (5.41)
	Vulvovaginal dryness	3	38.39 (12.34–119.43)	5.25 (0.44)	38.32 (108.58)	38.16 (12.27)
Respiratory, thoracic and mediastinal disorders	Dyspnoea	31	2.18 (1.53–3.11)	1.11 (0.54)	2.16 (19.4)	2.16 (1.51)
	Oropharyngeal pain	9	3.13 (1.63–6.03)	1.64 (0.45)	3.12 (13)	3.12 (1.62)
	Pulmonary hypertension	7	16.5 (7.85–34.68)	4.04 (1.46)	16.44 (101.33)	16.41 (7.81)
Vascular disorders	Pallor	7	8.27 (3.93–17.37)	3.04 (1.09)	8.24 (44.5)	8.23 (3.92)

Hepatic signals included ocular icterus (ROR: 76.90) and jaundice (ROR: 29.89). The current prescribing information notes jaundice and scleral icterus in the setting of acute hemolysis after abrupt discontinuation, and it contains a warning on hepatocellular injury observed at higher‐than‐labelled doses in another condition, recommending baseline and monthly liver tests during the first 6 months. Our FAERS findings may therefore represent an interplay between hemolysis, bilirubin clearance and liver susceptibility.

Neurocognitive terms such as brain fog (ROR: 10.06), disturbance in attention (ROR: 8.99) and memory impairment (ROR: 3.27) were disproportionately reported, although they are not part of the labelled safety profile. These events may reflect changes in neuronal energy balance and require further exploration. A serious unlabelled event, pulmonary hypertension (ROR: 16.50), was also detected, raising concern for possible vascular consequences of long‐term PKR modulation.

Reproductive events, including vulvovaginal dryness (ROR: 38.39) and menstrual disorder (ROR: 16.86), were observed in FAERS but are absent from the label. The prescribing information instead documents increases in testosterone and decreases in estrone and estradiol in men, reversible after discontinuation. The FAERS findings raise the hypothesis that hormonal regulation in women may also be affected, although further study is required.

Taken together, this FAERS‐based pharmacovigilance analysis outlines the real‐world safety profile of Mitapivat, identifying signals across hematologic, metabolic, hepatobiliary, neurologic, pulmonary and reproductive systems. While pivotal trials such as ACTIVATE and ACTIVATE‐T primarily reported transient and manageable laboratory abnormalities, our findings suggest a broader safety landscape that may have been underrecognized because of trial size, duration and eligibility restrictions [10].

Although most events were non‐serious, the emergence of pulmonary hypertension, hepatic manifestations and reproductive disturbances underscores the need for sustained pharmacovigilance and targeted clinical monitoring. These observations may be biologically interpreted within the pharmacological context of mitapivat, which allosterically activates red blood cell pyruvate kinase to restore glycolytic flux, increase ATP and lower 2,3‐diphosphoglycerate [11, 12]. In clinical development, increased serum urate was a consistent laboratory finding, possibly reflecting altered purine metabolism under conditions of enhanced cellular energy turnover, with catabolism mediated by xanthine oxidase contributing to uric acid accumulation [13, 14]. Hepatic manifestations such as ocular icterus and jaundice could be consistent with shifts in heme catabolism and bilirubin processing at the level of conjugation and transport, involving UGT1A1 as well as canalicular export proteins such as MRP2 and the bile salt export pump (BSEP) [15]. Signals of iron‐handling abnormalities may align with erythropoietic stimulation that suppresses hepatic hepcidin through erythroferrone, thereby increasing intestinal iron absorption and macrophage iron release, a process dependent on ferroportin activity at the basolateral membrane [16, 17]. Neurocognitive complaints could be interpreted within broader links between systemic energy metabolism and neuroglial coupling, notably via the astrocyte‐neuron lactate shuttle (ANLS) mechanism, which is posited to support neuronal ATP homeostasis under metabolic stress. These mechanistic links remain largely hypothetical in the context of mitapivat‐associated neurocognitive events [18, 19]. Taken together, these mechanistic considerations illustrate biologically coherent pathways through which mitapivat‐associated metabolic modulation could manifest as clinically relevant toxicities, highlighting the need for validation in prospective studies incorporating molecular biomarkers.

In conclusion, FAERS‐based pharmacovigilance provides a valuable tool for risk identification after drug approval. Although causality cannot be established, the identified signals generate hypotheses that require mechanistic and epidemiologic confirmation. Prospective studies integrating metabolic, hematologic and hormonal biomarkers with clinical surveillance will be essential to disentangle drug‐related mechanisms from background comorbidities. Clinicians should remain attentive to potential adverse events, especially those absent from the current prescribing information, as Mitapivat use becomes more widespread in clinical practice.

## Author Contributions

Conception and design: Qiong Liu. Collection and assembly of data: Guojun Liang. Data analysis and interpretation: Guojun Liang. Manuscript writing: All authors. Final approval of manuscript: All authors.

## Disclosure

During the preparation of this work, the authors used DeepL in order to improve readability. After using this tool/service, the authors reviewed and edited the content as needed. The authors take full responsibility for the content of the publication.

## Ethics Statement

The authors have nothing to report.

## Conflicts of Interest

The authors declare no conflicts of interest.

## Supporting information


**Figure S1:** jcmm70893‐sup‐0001‐FigureS1.docx.

## Data Availability

The data related to this study has been stored in a publicly accessible repository (https://fis.fda.gov/extensions/FPD‐QDE‐FAERS/FPD‐QDE‐FAERS.html). All data produced or examined in this article are available within the published paper and its Supporting Information.
