# Supplementary material for: Mitapivat‐Associated Adverse Effects and Potential Mechanistic: Insights From Real‐World Data
Source: J Cell Mol Med. 2025 Oct 13;29(19):e70893. doi: 10.1111/jcmm.70893 (PMC12516351; doi:10.1111/jcmm.70893)

Supplementary Figure 1. Time to onset of Mitapivat-associated adverse events reported to FAERS (Q1 2022-Q2 2025). Median onset was 134 days, indicating that many events emerged after prolonged exposure.


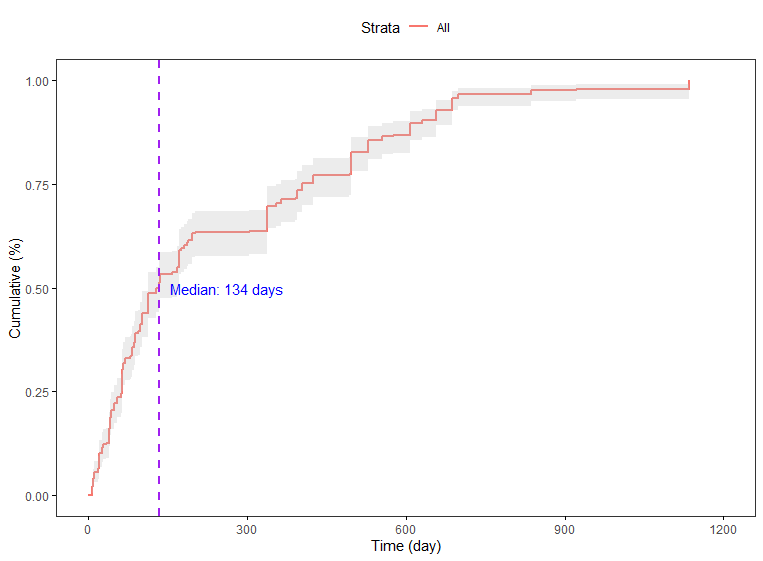

Supplement: Supplementary file 1 — Figure S1: jcmm70893‐sup‐0001‐FigureS1.docx. [file JCMM-29-e70893-s001.docx]
